# Globularity and language-readiness: generating new predictions by expanding the set of genes of interest

**DOI:** 10.3389/fpsyg.2014.01324

**Published:** 2014-11-25

**Authors:** Cedric Boeckx, Antonio Benítez-Burraco

**Affiliations:** ^1^Catalan Institute for Advanced Studies and Research (ICREA)Barcelona, Spain; ^2^Department of Linguistics, Universitat de BarcelonaBarcelona, Spain; ^3^Department of Spanish Philology and its Didactics, University of HuelvaHuelva, Spain

**Keywords:** globularity, language-ready brain, thalamus, cortex, basal ganglia, cerebellum, biolinguistics, vocal learning

## Abstract

This study builds on the hypothesis put forth in Boeckx and Benítez-Burraco (2014), according to which the developmental changes expressed at the levels of brain morphology and neural connectivity that resulted in a more globular braincase in our species were crucial to understand the origins of our language-ready brain. Specifically, this paper explores the links between two well-known ‘language-related’ genes like *FOXP2* and *ROBO1* implicated in vocal learning and the initial set of genes of interest put forth in Boeckx and Benítez-Burraco (2014), with *RUNX2* as focal point. Relying on the existing literature, we uncover potential molecular links that could be of interest to future experimental inquiries into the biological foundations of language and the testing of our initial hypothesis. Our discussion could also be relevant for clinical linguistics and for the interpretation of results from paleogenomics.

## OVERVIEW

A central goal of the biolinguistic enterprise (Di Sciullo and Boeckx, 2011) is to shed light on the genetic basis of a complex trait, characteristic of modern humans, which we dub the language-ready brain, understood as the set of neural structures that serve as a critical biological substrate for the acquisition and use of grammatical systems routinely called natural languages.

In this paper we would like to expand on the hypothesis we put forth in Boeckx and Benítez-Burraco (2014), taking into account considerations that we set aside when we formulated our original claim and constructed the initial set of genes we took to be of interest. This hypothesis, in a nutshell, amounted to claiming that the developmental changes expressed at the levels of brain morphology and neural connectivity that resulted in a more globular braincase in our species (‘globularity’) were crucial to understand a central aspect of the language-readiness of the modern human brain, viz. the ability to form complex, cross-modular thoughts. Using terminology common among linguists, we can say that this aspect of language pertained to the syntax-semantics interface. As we pointed out at the outset of our paper, this facet of our language-ready brain is distinct from another ability that is equally central to the acquisition and use of language: the ability to communicate these thoughts to conspecifics (“morpho-phonology” for linguists). In the technical literature (Berwick et al., 2013), this ability is said to be the responsibility of an ‘externalization’ component. The latter is by far the best studied aspect of our language-ready brain, as it can be related most directly to the vocal learning abilities manifested in other species, about which we are beginning to know a fair amount. The externalization component is also the one that most obviously breaks down in a number of language disorders. Thanks to this, it has been possible to begin to relate its functioning to some genes, most notably *FOXP2* (Lai et al., 2001; Fisher and Marcus, 2006; Graham and Fisher, 2013).

Our aim here is to examine possible connections between the set of genes considered in Boeckx and Benítez-Burraco (2014) and what we know about the molecular substrate that allows humans to externalize the complex thoughts that our language-ready brain allows us to form. One particular reason to suspect that such connections are worth examining is that our initial investigation already revealed points of contact between our core set of genes (*USF1*, *RUNX2*, *DLX1*, *DLX2*, *DLX5*, *DLX6*, *BMP2*, *BMP7*, and *DISP1*) and *FOXP2* and its partners. In addition, the anatomical structure we focused on in our 2014 paper, the thalamus, not only plays a crucial role in establishing and maintaining efficient cortico-cortical connections that we deemed necessary for cross-modularity (Boeckx and Benítez-Burraco, 2014: see Results and Discussion; Wang et al., 2010a; Buckner and Krienen, 2013), it also functions as a bridge between the cortex and the subcortical structures most frequently associated with the externalization component of language (the basal ganglia and the cerebellum). The fact that it has long been suspected that the *FOXP2* network appears to play a critical role in the establishment and maintenance of these neural circuits (Vargha-Khadem et al., 2005; Fisher and Marcus, 2006; Kurt et al., 2012) suggest to us that it makes sense to look for potential connections. Thinking of Darwin’s (1871) concept of ‘correlated growth,’ we ought to expect changes at the level of the thalamus to affect the externalization component as much as the syntactic-semantic aspects of language that were our original focus, especially given the fact that unlike other species, our linguistic mode of communication appears to be able to express virtually all the thoughts we can construct. Thus, the present paper can be seen as an attempt to extend the scope of our initial study.

Much like we claimed that the roots of cross-modularity were quite ancient in our (2014) piece, we also think that the externalization component of the language-ready brain rests on solid evolutionary foundations. Although we believe that our nearest (extinct) relatives differed from us in certain ways that pertain not only to the syntax-semantics interface but also to the domain of externalization, we think that they were almost certainly capable of vocal learning, and had built a niche that favored communication and cooperation. Dunbar (1996), Deacon (1997), Mithen et al. (2006), Wrangham (2009), and Tomasello (2014), contain detailed arguments in favor of our communicative abilities and the contexts in which communication takes place having ancient roots: our (extinct) ancestors were highly social, cooperative, and undoubtedly displayed symbolic practices (rituals). They were big-brained, and displayed many anatomical and even molecular signs of vocal learning (Dediu and Levinson, 2013). In addition, they were skillful tool-users, and displayed some modern-like brain structures that people have long associated with aspects of language, such as a well-developed Broca’s area, and lateralization patterns reminiscent of ours (Frayer et al., 2010). All of these properties were unquestionably important for linguistic communication as we know it to ultimately develop. Thus we find ourselves in general agreement with the many researchers who treat Neanderthals as vocal learners, but we also side with scholars like Lieberman (2007), who has long insisted on modern speech abilities requiring an anatomy specific to modern humans. For us, this specific anatomy boils down to globularity, the outcome of a species-specific developmental trajectory that takes during the first year of life (Boeckx and Benítez-Burraco, 2014, and references therein), although some of its consequences only manifest themselves after a certain amount of maturation. The view we defend here is that globularity most likely entailed changes that led to some additional, possibly selected-for, adjustments within molecular pathways that were involved in vocal learning and were recruited to give rise to speech proper. In this respect, our stance fits well with the ‘musical protolanguage’ scenario first proposed by Darwin (1871) and recently refined by several authors (Mithen et al., 2006; Fitch, 2010). (Before proceeding, it may be worth mentioning that instead of speech, we should perhaps talk about sensori-motor abilities to capture the fact that the vocal channel is but one mode of linguistic externalization. Ultimately, signed languages must be integrated into our theorizing. Having said this, we will mostly discuss draw from the literature on vocal learning in what follows, if only because this is where progress in comparative cognition has been most obvious.)

The literature on vocal learning has been growing steadily over the last 10 years, and enables us to ground our proposal onto well-established anatomical and molecular findings. It is not our goal here to provide an exhaustive review of the literature, as several such reviews already exist. For example, Fitch and Jarvis (2013) provide numerous references in support of neural pathways necessary for vocal learning in birds, and by analogy in humans. One such pathway, the so-called posterior pathway, necessary to produce song/speech, provides a direct forebrain control of brainstem vocal motor neurons, lacking in vocal non-learners (Fitch, 2010). Another pathway, the so-called anterior pathway, is not necessary for producing songs, but is necessary for learning songs and for modifying already learned songs. This pathway consists of a loop linking basal ganglia (especially, the striatum), the thalamus, and (for humans) cortical structures (Miller and Buschman, 2007). Among the cortical structures, one finds the well-known fronto-temporal network connecting Broca’s and Wernicke’s areas (Hickok and Poeppel, 2007 and much subsequent work refining the classical Broca–Wernicke–Lichtheim–Geschwind model presented in all textbooks on this topic; in particular; Hickok et al., 2011; Hickok, 2012). Although traditionally taken to constitute ‘the language network,’ the fronto-temporal connections are best regarded implicated in aspects of the externalization component of language (see Boeckx et al., 2014, for review).

Our focus here will not be so much on these neuroanatomical findings, but rather on some of the most prominent molecular signatures of vocal learning identified in the literature. Besides *FOXP2* and its partners (*FOXP1*, *FOXP4*, *CNTNAP2*, etc.), enriched gene regulation of the ROBO/SLIT family of genes has been identified (Fitch and Jarvis, 2013). Both *FOXP2* and *ROBO1* have been associated with language/speech disorders (specific language impairment and dyslexia, respectively; see below), and therefore strike us as valuable starting points in our quest. Recent studies, such as Kato et al. (2014), also use these genes as probes with the same confidence we want to use them here. We stress, though, that taking *FOXP2* and *ROBO1* as focal points does not mean that other “language-related” genes found in the literature are less important (see Benítez-Burraco, 2009, for a comprehensive survey). It is just that the potential functional links discussed below appear to us to be the most promising ones at this point, in large part because, as already mentioned above, these genes and their partners have been consistently related to brain areas and neural circuits that have been repeatedly implicated in the externalization of language. In part, our choice was also dictated by the fact that in most cases, and in contrast to *FOXP2* and *ROBO1*, the genes related to language have not yet given rise to a solid body of knowledge on which we could rely. We certainly hope that future work will complement our perspective, which at this point is but a small corner of a much bigger puzzle.

## METHODS, AIMS, AND LIMITATIONS

As already stated, our goal in this paper is to expand the gene list that potentially fall under the scope of our initial hypothesis, and looking for potential functional connections with the initial gene set discussed in Boeckx and Benítez-Burraco (2014). To do so, we have extensively reviewed the literature on both the *FOXP2* and *ROBO1* interactomes and functional networks, as presently known, and examined connections with the network put forth in our (2014) paper.

Our *modus operandi* has been as follows:

(a) We used PubMed^1^ to identify potential partners of the genes of interest in our (2014) paper, *FOXP2* and *ROBO1*. We narrow our search by using key terms of interest for us, including (but not restricted to) “brain,” “cognition,” “language,” “syntax,” “semantics,” “phonology,” “speech,” or “vocal learning.”
(b) We also searched the literature via PubMed looking for genes related to clinical conditions and symptoms of interest for us. Some of the key search terms used were: “language disorder,” cognitive disorder,” “intellectual disability,” “syntax deficit,” “semantic deficit,” “phonological deficit,” “speech deficit,” “dyslexia,” “schizophrenia,” “autism,” “autism spectrum disorder,” etc. When a gene of interest was identified, we looked for potential links with our core set of genes, refining the searching process by adding the name of genes of interest previously identified.
(c) We also used PubMed to look for genes related to brain areas, circuits, neural processes, neurotransmitters, etc. of interest for us; specifically, the neurological considerations of Boeckx and Benítez-Burraco (2014) as well as those neural substrates highlighted in the vocal learning and *FOXP2* literature. For this, we used search terms like “thalamus,” “thalamo-cortical connection,” “motor cortex,” “neurite outgrowth,” etc.
(d) We did not systematically gather information beyond more than three connection levels. Thus, our limit was of the sort ‘*ROBO1* is connected to X which is in turn connected to Y.’ We considered additional levels only if the identified genes were more closely connected to some other gene(s) of interest previously reviewed. For example, ‘*ROBO1* is connected to X which is in turn connected to Y which is in turn connected to Z (but Z is connected to *FOXP2*).’
(e) We explored potential connections with genes selected in anatomically modern humans (AMH; Pääbo’s 2014 list).

Because the amount of literature on single genes varies greatly, we did not necessarily discard any gene because the number of manuscripts was under a value we may have pre-selected.

In addition to PubMed, we also relied on the following databases, which we also used for our (2014) paper.

(1) the microarray database of the Allen Brain Atlas^2^.
(2) the Prenatal LMD Microarray search engine^3^.
(3) the Developmental Transcriptome browser^4^ of the Allen Brain Atlas.
(4) OMIM for the linguistic and cognitive deficits linked to the mutation of genes of interest^5^.

Additionally, we have exploited the information provided in Kuhlwilm et al. (2013), where 691 genes were found to be differentially expressed after *RUNX2* transfection in neuroblastomic SH-SY5Y cells, and we have studied the overlap with the FOXP2 targets list provided in Konopka et al. (2009), who also relied on this cell line (see **Table 1**). [Other comprehensive lists of FOXP2 targets we also took into account are Spiteri et al. (2007) and Vernes et al. (2007).] We have also examined the overlap between the list provided in Kuhlwilm et al. (2013) and the list of differentially expressed genes in vocal learners in Wang (2011; see **Table 2**). Finally, we have explored links and predicted interactions generated by String 9.1^6^. String 9.1 predicts direct/physical and indirect/functional associations between proteins that derive from four sources: genomic context, high-throughput experiments, conserved coexpression, and the knowledge previously gained from text mining (Szklarczyk et al., 2011). For each new candidate gene, we did an extensive literature survey to confirm its viability as a member of our network. The next section summarizes our results.

**Table 1:** Overlap between the FOXP2 targets list provided in Konopka et al. (2009) and the 691 genes found to be differentially expressed after *RUNX2* transfection in neuroblastomic SHSY5Y cells in Kuhlwilm et al. (2013).

Gene					
*CDCA7L*
*CEBPB*
*CHRM3*
*CXCR4*
*DPYSL3*
*ECT2*
*EFNB2*
*EGR1*
*FRAS1*
*FRMD3*
*FSTL5*
*IGFBP3*
*KCNT1*
*LIG1*
*NCAM1*
*NNAT*
*PI15*
*PPP2R2B*
*PRKCA*
*PTRF*
*RBL1*
*RET*
*RUNX1T1*
*SCARA3*
*STC2*
*STMN4*
*TMPO*
*TMTC2*

**Table 2:** Overlap between the list of differentially expressed genes in vocal learners in Wang (2011) and the list of genes found to be affected by *RUNX2* overexpression in 10 different human cell lines in Kuhlwilm et al. (2013).

Gene					
*C1ORF116*
*CASP8(AP2)*
*CEP192*
*EFCAB7*
*PARP1*
*PLEKHH1*
*PPL*

## RESULTS AND DISCUSSION

**FIGURE 1:**
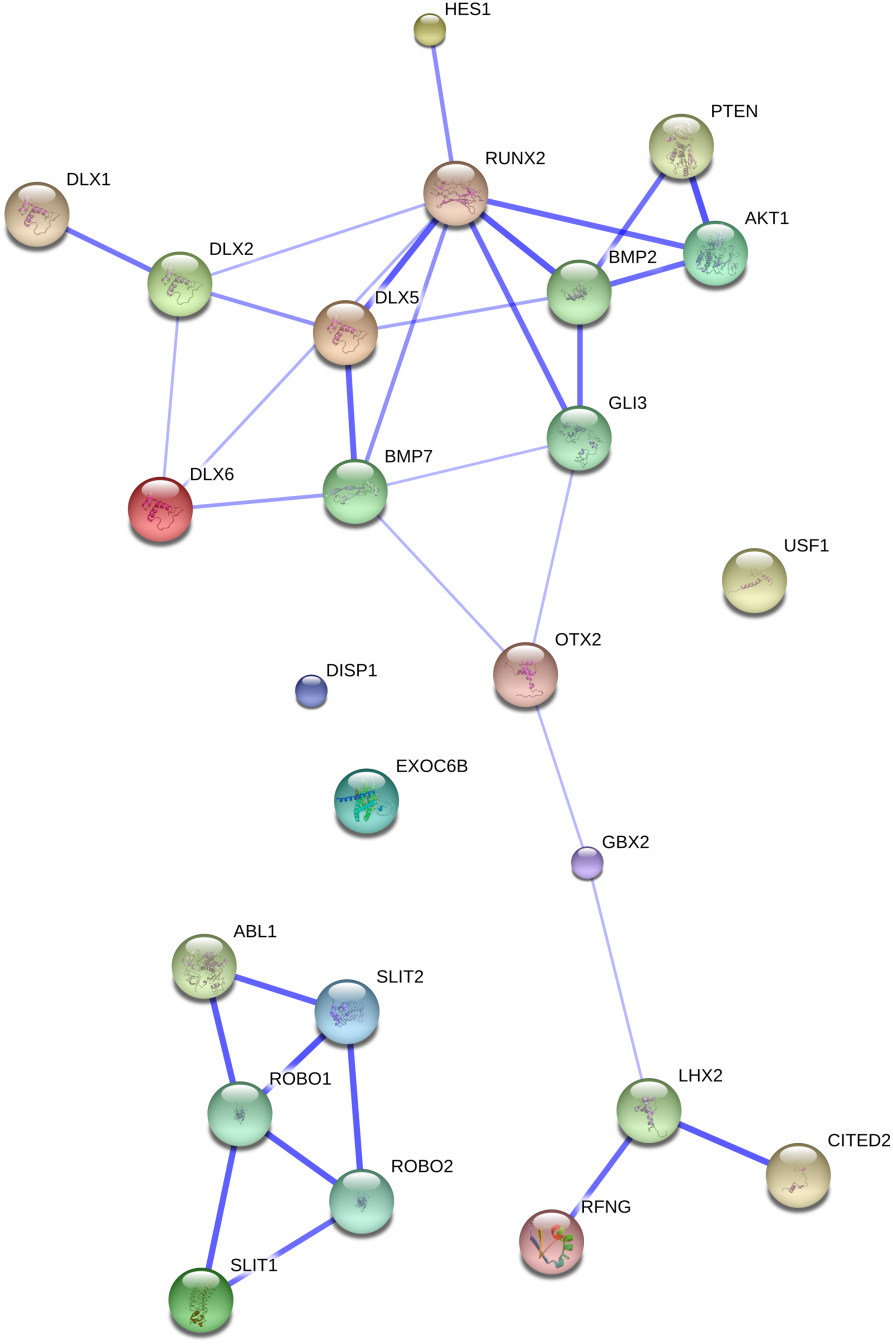
**The ROBO/SLIT suite and the genes related to our initial gene set.** The network was generated by String 9.1 with the proteins discussed in Section “The ROBO/SLIT Suite” plus the gene set related to globularity as advanced in Boeckx and Benítez-Burraco (2014). The medium confidence value was 0.0400. Nodes representing the proteins encompassing the network are colored randomly. In this confidence view, stronger associations between proteins are represented by thicker lines. The figure does not represent a fully connected graph, but readers are asked to bear in mind that String 9.1 predicts associations between proteins that derive from a limited set of databases. The material discussed in the main text lead us to suspect connections that String does not generate (although we wish to note that just adding a few genes, not discussed in this paper, yield a connected graph). It should be emphasized that the nature of String 9.1 is essentially predictive, and not explanatory. Although we have confirmed all the links we discuss here in the literature, they need to be confirmed at the brain level and in relation to language. Additionally, the diagram only represents the potential connectivity between the involved proteins, but this has to be mapped onto particular biochemical networks, signaling pathways, cellular properties, aspects of neuronal function, or cell-types of interest that can be confidently related to aspects of language development and function.

**FIGURE 2:**
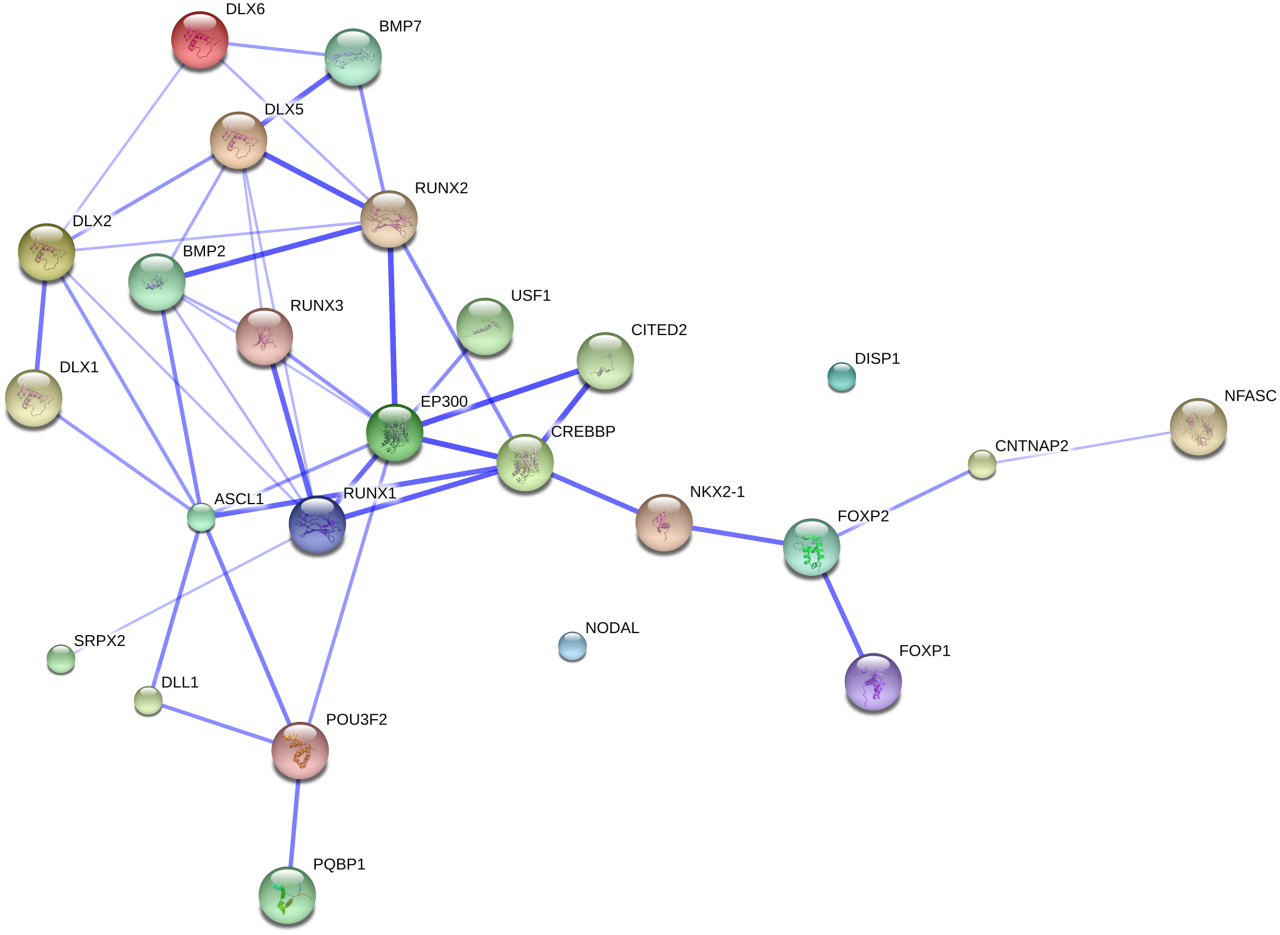
**The FOXP2 suite and the genes related to globularity.** The network was generated by String 9.1 with the proteins discussed in Section “*FOXP2* and Partners” plus the gene set related to globularity as advanced in Boeckx and Benítez-Burraco (2014). The medium confidence value was 0.0400. The caveats noted for **Figure 1** apply.

All the genes discussed in this section were selected as a result of the search method described in Section “Methods, Aims, and Limitations.” The information we provide about each gene pertains to the potential connections with the genes of interest in Boeckx and Benítez-Burraco (2014), with special emphasis on brain growth and neural interconnection. We also report on whether the gene is known as candidate for a clinical condition associated with a variety of linguistic, cognitive, and cranio-facial deficits (results from linkage or association analyses, GWAS, or the discovery of point mutations or chromosomal rearrangements affecting the gene function). We begin with *ROBO* genes and their partners (see The ROBO/SLIT Suite) (see **Figure 1**), then turn our attention to *FOXP2* and its partners (see *FOXP2* and Partners) (see **Figure 2**), and conclude this section by highlighting other related genes of interest (see Other Genes of Interest).

### THE ROBO/SLIT SUITE

Members of the *ROBO* gene family, which includes *ROBO1* and *ROBO2*, discussed here, play an important role to control the development of ascending or descending major axon tracts to or from the forebrain, and interneuron migration in the forebrain, through interactions with the ligands SLIT1, SLIT2, and SLIT3 (Andrews et al., 2006; López-Bendito et al., 2007; Dugan et al., 2011).

#### ROBO1

*ROBO1* encodes an axon guidance receptor to navigate the growth of longitudinal axons (Devine and Key, 2008). Mutations in *ROBO1* are associated with human dyslexia and speech sound disorder (Hannula-Jouppi et al., 2005; Mascheretti et al., 2014). As Wang (2011) notes, *ROBO*s/*SLIT*s show differential expression in song nuclei of songbirds. The avian arcopallium contains large regions with parallels to the mammalian cortex. Particularly, the robust nucleus of the arcopallium (RA) projection neurons are proposed to be analogous to the layer V neurons of the facial motor cortex of mammals that sends direct projections to the brainstem vocal nucleus in humans (Jarvis, 2004), while the arcopallium surrounding the RA may be analogous to the layer V neurons of mammalian motor cortex (Feenders et al., 2008). Wang (2011) detected the expression of all *ROBO*s and *SLIT*s in the RA and the surrounding arcopallium in the adult male zebra finch brain. As a comparison, three regions (layer V, entorhinal cortex and piriform cortex) of the adult rat cortex are found to express all five *ROBO*s/*SLIT*s genes (Marillat et al., 2002). Of them, only layer V contains the descending motor pathway neurons. Thus, these findings seem to support the analogy between the RA surrounding arcopallium to the motor cortex (layer V) of mammals.

Additionally, Wang (2011) found specialized expression patterns of *ROBO1*, *SLIT1*, and *ROBO2* (but not *SLIT2* and *SLIT3*), in the RA of adult male zebra finch, relative to the surrounding arcopallium. Only *ROBO1* is up-regulated in the RA, while *ROBO2* and *SLIT1* are down-regulated in this region. This difference between *ROBO1* and *ROBO2* in the RA is remarkable, since the two *ROBO*s typically act together for axon guidance in the forebrain (López-Bendito et al., 2007) and in rats, they are co-expressed in most telencephalic regions (Marillat et al., 2002). For birds, Wang (2011) suggests that *ROBO1* is co-opted in the specialized vocal motor output nucleus of vocal learning birds, but admits that it remains to be seen whether it participates in forming the unique direct projections to the brainstem vocal nucleus. Wang (2011) also considers worth exploring the *ROBO1* expression patterns in the analogous face motor cortex area in human and other mammalian vocal learners or non-learners. In this context, Wang (2011) observes that recent studies showed that a splice variant of *ROBO1*, called ROBO1a, is highly enriched in the temporal auditory neocortex and/or temporal association neocortex, while ROBO1b is enriched in the prefrontal neocortex where face motor cortex and Broca’s area develops (Johnson et al., 2009). Both *ROBO1* and *ROBO2* are specifically involved in thalamocortical axons (TCA) development (López-Bendito et al., 2007; Marcos-Mondéjar et al., 2012). TCAs represent the major input to the neocortex and modulate cognitive functions, consciousness, and alertness.

#### ROBO2

*ROBO2* is a putative candidate for autism (Suda et al., 2011). Moreover, a polymorphism near the gene has been associated with expressive vocabulary growth in the normal population (St Pourcain et al., 2014). The *locus* for the gene is also a linkage region for dyslexia (Fisher et al., 2002) and speech-sound disorder and reading (Stein et al., 2004). *ROBO2* is a functional partner of *DLX1* and *DLX2* too, two of the genes we highlighted in the context of our ‘globularity and language-readiness hypothesis.’ In particular, both Dlx1 and Dlx2 factors are required to promote tangential migration to the olfactory bulb via Robo2 (Long et al., 2007).

#### SLIT2

ROBO’s ligands SLITs are also worth considering in the context of our hypothesis. During human brain evolution, considerable enlargement of the association areas of the cortex is accompanied by a comparable enlargement of corresponding association thalamic nuclei in the diencephalon, but developmental mechanisms coordinating these expansions remain unknown. SLITs proteins have been claimed to play important roles in brain development before and after birth, and several pieces of evidence suggest that they may be involved in species-specific developmental patterns of the thalamus. Specifically, Slit2 has been hypothesized to act as a repellent for migrating sub-ventricular zone cells (Hu, 1999; Chen et al., 2001; Wu et al., 2001). The Slit/Robo guidance family also ensures the restraint of corticothalamic axons within the internal capsule, and upon reaching the diencephalon–telencephalon boundary, direct them dorsally toward the thalamus rather than crossing the midline (Bagri et al., 2002; López-Bendito et al., 2007; Braisted et al., 2009; Leyva-Díaz and López-Bendito, 2013). *Slit1* and *Slit2* are expressed in overlapping domains including the ganglionic eminences, prethalamus, hypothalamus, and the germinal zone of the thalamus (Bagri et al., 2002); while *Robo1* and *Robo2* are expressed in complementary patterns in the cortical plate, intermediate zone, and thalamus (López-Bendito et al., 2007).

*Slit2* is also strongly expressed in the region of the cortical hem, a boundary region that has been shown to express transcripts of members of the patterning gene families Wnt, Bmp, and Msx, as well as Shh (see Grove et al., 1998), genes linked to our initial (2014) gene set of interest. Specifically, the midline repellent Slit2 orients migration of corridor neurons and thereby switches thalamic axons from an external to a mammalian-specific internal path. Bielle et al. (2011) reveal that subtle differences in the migration of conserved intermediate target neurons trigger large-scale changes in thalamic connectivity, and opens perspectives on Slit functions and the evolution of brain wiring. In particular, this suggests that *SLIT2* may be involved in the human specific pattern of DLX-expressing interneuron migration from the ganglionic eminence into the thalamus (Letinic and Rakic, 2001), which is held to be responsible for the human-specific enlargement of higher-order thalamic nuclei like the mediodorsal nucleus or the pulvinar, structures highlighted by Boeckx and Benítez-Burraco (2014) in the context of globularity and language-readiness. It is also worth pointing out here that the promoter region of *SLIT2* has been claimed to have been under significant positive selection in humans relative to other primates (Haygood et al., 2007).

#### SLIT1

Interestingly, another SLIT gene, *SLIT1*, an effector of ROBO1, serves a direct downstream target for the speech-language related gene *FOXP2* (Vernes et al., 2007; Konopka et al., 2009). Moreover, three genes that encode proteins that are part of the centrosome assembly that interacts with SLIT1, necessary for proper neuron migration and neural process extension, show differential expressions among (mammalian) vocal learners (Wang, 2011): *CKAP5*, *PCM1*, and *CEP192* (Higginbotham et al., 2006).

#### HES1

*HES1* is functionally related to *ROBO1*. During neurogenesis the transcriptional activation of *Hes1* is a key step for the Slit/Robo signaling pathway that modulates the transition between primary and intermediate progenitors (Borrell et al., 2012). *Hes1* is also a direct interactor of runx2 (Suh et al., 2008), one of the most relevant genes discussed in Boeckx and Benítez-Burraco (2014) in the context of globularity. *Hes1* is expressed in the ventral posterior part of the thalamus (Lein et al., 2007), and the HES1 pathway is related to language development and craniofacial development. Thus, the reduced expression of *EXOC6B* affects the expression of *HES1* (Wen et al., 2013). In turn, *EXOC6B* haploinsufficiency has been related to intellectual disability, language delay, facial asymmetry, and vertebral and/or craniofacial abnormalities (Wen et al., 2013). Importantly, *Hes1* is involved in the development of both GABAergic neurons, whose relevance we discussed in our (2014) paper, and of dopaminergic neurons, routinely mentioned in the literature on motor behavior and vocal learning in particular. According to Long et al. (2013), *Hes1* silencing is able to promote bone marrow mesenchymal stem cells to differentiate into GABAergic neuron-like cells *in vitro*. In particular, it has been proposed that *Hes1* plays a prominent role in regulating the location and density of mesencephalic dopaminergic neurons (Kameda et al., 2011). In *Hes1* homozygous null mutant mice the lack of the gene results in disturbances in the inductive and repulsive activities of the isthmic organizer and leads to the failure of cranial neurulation due to the premature onset of neural differentiation.

#### GBX2 and its partners: LHX2, GLI3, and OTX2

Another gene of relevance that controls the expression of the Robo suite and is essential for TCA development is *GBX2*. In Gbx2-deficient mice TCAs are abolished. Specifically, thalamic axons are mostly misrouted to the ventral midbrain and dorsal midline of the diencephalon (Chatterjee et al., 2012). Gbx2 acts through three different mechanisms: first, Gbx2 and Lhx2 compete for binding to the *Lmo3* promoter and exert opposing effects on its transcription; second, repressing *Lmo3* by Gbx2 is essential for Lhx2 activity to induce *Robo2*; and third, Gbx2 represses *Lhx9* transcription, which in turn induces *Robo1*.

Lhx2 affects the topographical sorting of axons by directly regulating the expression of *Robo1* and *Robo2* (Marcos-Mondéjar et al., 2012). Overexpression of *Lhx2* gives rise to defective TCA guidance *in vivo*, while its conditional deletion in the thalamus alters projections from the medial geniculate nucleus and from the caudal ventrobasal nucleus (Marcos-Mondéjar et al., 2012). *Lhx2* is also involved in thalamic development under the control of Shh, encoded by another member of our (2014) gene set. Specifically, low Shh signaling induces *Lhx2* expression, as well as *Gbx2* expression, in the caudal thalamus (Barth and Wilson, 1995; Hashimoto-Torii et al., 2003; Kiecker and Lumsden, 2004; Scholpp et al., 2006; Szabó et al., 2009; Vue et al., 2009), thus specifying thalamic neuronal subtype identities within this region (Scholpp and Lumsden, 2010). Additionally, *Lhx2* (together with *Lhx9*) emerges as a crucial factor driving neurogenesis and maintaining the regional integrity of the caudal forebrain. Moreover, Lhx2-mediated neurogenesis seems to be involved in maintaining the integrity of cortex and regulating cortical size (Chou and O’Leary, 2013).

Interestingly, another of LHX2 interactors, namely, RFNG, also exhibits a fixed change (Y281H) in humans. Ectopic expression of *Lhx2* induces *Rfng* expression in chicken (Rodríguez-Esteban et al., 1998). Importantly, *rfng* encodes an *O*-fucosylpeptide 3-β-*N*-acetylglucosaminyltransferase which negatively modulates Notch signaling in postmitotic neurons by inhibiting *Hes1* expression in primary neurons (Mikami et al., 2001). In zebrafish *rfng* is required as well for *wnt1* expression at hindbrain boundaries and contributes to the regulation of cell differentiation (Amoyel et al., 2005). Finally, LHX2 is a functional partner of CITED2 (Glenn and Maurer, 1999). As already mentioned in Boeckx and Benítez-Burraco (2014), *CITED2* is an interesting gene for us, as it plays a key role in brain/skull development (as one of RUNX2 partners), in thalamus growth (as a partner of LHX2), and in the development of subcortical structures. We return to this gene in the next section, where we discuss its role as FOXP2 partner.

Going back to *Gbx2*, it is worth noting that *Gbx2* expression is highly reduced in *Gli3* mutants, in which the medial and intralaminar nuclei of the thalamus are specifically and severely affected (Haddad-Tóvolli et al., 2012). Like Lhx2, Gli3 interacts with Shh during thalamic development (Haddad-Tóvolli et al., 2012). Moreover, Gli3 regulates calvarial suture development by controlling Bmp-Smad signaling, which integrates a Dlx5/Runx2-II cascade (Tanimoto et al., 2012). Mutations in *GLI3* have been found in people affected by Greig cephalopolysyndactyly syndrome, a condition in which craniosynostosis is an important feature (Debeer et al., 2003). Interestingly, most (∼98%) of Altaic Neanderthals and Denisovans had a different sequence in *GLI3* compared to AMHs. While the latter retained the ancestral sequence, the former gained a non-synonymous change that appears to be mildly disruptive (Castellano et al., 2014).

Finally, Gbx2 interacts with Otx2 to determine the midbrain-hindbrain boundary in vertebrates. Moreover, both *Gbx2* and *Otx2* play a key role in the development of the cerebellum, a key brain component of vocal learning. Specifically, both genes are important during early neurogenesis and regulate the positioning and formation of the cerebellar primordium (Hashimoto and Hibi, 2012). In Boeckx and Benítez-Burraco (2014) we reviewed the link between *OTX2* and language in connection with the *FOXP2* interactome. Interestingly, a human-specific conserved deletion (hCONDEL) occurres downstream *OTX2* (McLean et al., 2011). Of more direct interest is the fact that Gbx2 restricts *Otx2* expression to forebrain and midbrain, competing with class III POU factors (Inoue et al., 2012). One of these factors is encoded by *POU3F2*, a target of FOXP2 (Konopka et al., 2012), to which we return below.

#### ABL1

*ABL1* provides another link to *ROBO1* that we find of interest in the context of our hypothesis. *ABL1* encodes a cytoplasmic and nuclear tyrosine kinase that seems to play a prominent role in cell differentiation, division, and adhesion (see Matsumura and Toyoshima, 2012, for a recent review). Both *ROBO1* and *ABL1* show a differential expression profile in the hippocampus of schizophrenics (Benes et al., 2009). *ABL1* is expressed in the thalamus too. In rats Abl1 levels are reduced specifically by safety learning but not fear conditioning, suggesting that *Abl1* may be involved in the regulation and/or the activation of specific auditory networks within the thalamus (Habib et al., 2013). Moreover, high levels of ABL1 have been detected as well in the brains of people suffering from Parkinson (Hebron et al., 2013). Interestingly, *Abl1* is also highly expressed in bone tissue in newborn mice and osteoblasts in the fetus. Mice homozygous for mutations in *Abl1* display foreshortened crania (Schwartzberg et al., 1991) and are osteoporotic because of having dysfunctional osteoblasts (Li et al., 2000). Finally, *ABL1* affects, via BMP signaling, the differentiation of cranial neural crest cells and the induction of myogenic cell proliferation in the cranial mesoderm during tongue development (Iwata et al., 2013). *BMP*s are among the genes we took to be central in our (2014) paper.

#### AKT1

*AKT1* is another promising gene for us in the context of the ROBO suite, and the genes we discussed in our (2014) piece. Akt1 enhances transcriptional activity of *Runx2* in mice (Fujita et al., 2004). *AKT1* encodes a serine-threonine protein kinase. In the developing cerebellum AKT1 is a critical mediator of growth factor-induced neuronal survival (Dudek et al., 1997). In mice mutations affecting both *Akt1* and *Akt2* cause impairment of bone formation (Peng et al., 2003). In humans, mutations in *AKT1* have been associated with the Proteus syndrome (Cohen, 2014), a condition similar to gigantism in which macrocephaly is a prominent symptom (Lindhurst et al., 2011). Moreover, *AKT1* is a susceptibility gene for schizophrenia. According to Emamian et al. (2004) a significant association exists between schizophrenia and an *AKT1* haplotype associated with lower AKT1 protein levels; moreover, a greater sensitivity to the sensorimotor gating-disruptive effect of amphetamine is conferred by AKT1 deficiency.

#### PTEN

Finally, PTEN is an effector of AKT1: in mice Pten is required for a robust depletion of nuclear phosphorylated Akt1 (Mistafa et al., 2010). *PTEN* is another candidate for Proteus syndrome (Lindhurst et al., 2011), but also for autism with macrocephaly (Buxbaum et al., 2007). Autism syndrome disorder subjects with *PTEN* mutations and reduced PTEN protein levels show strong reductions in working memory and processing speed, resulting from white-matter abnormalities (Frazier et al., 2014). Interestingly, in mice *Pten* is repressed by Hes1 as part of the Notch signaling pathway (Wong et al., 2012). Together with *Hes1* (and other genes like *Socs1* or *Stat3*), Pten comprisses a functional network that plays an important role in the control of the fate of ependymal stem progenitor cells of the spinal cord in response to motoneuron degeneration (Marcuzzo et al., 2014). Hes1, Notch1, Akt1, and Pten are functionally related (Palomero et al., 2008).

### *FOXP2* AND PARTNERS

Of all the language-related genes identified to date, none is as well characterized as *FOXP2* (Graham and Fisher, 2013; French and Fisher, 2014). We begin this section by all too briefly summarizing some of the major facts about the gene. *FOXP2* encodes a transcription factor that mostly works as a gene repressor (Shu et al., 2001). In the human brain it is expressed in several areas, including layer VI of the cortex, the thalamus, the cerebellum, and the basal ganglia (Ferland et al., 2003; Takahashi et al., 2003). The FOXP2 protein seemingly contributes to the development and function of cortico-thalamic-striatal circuits supporting motor planning, sequential tasks, and procedural learning (see Vargha-Khadem et al., 2005; Fisher and Scharff, 2009 for reviews). Recently, it has been suggested that the human variant may help achieve a faster transition between declarative and procedural learning systems (Schreiweis et al., 2014). Mutations in *FOXP2* cause speech and language deficits (Vargha-Khadem et al., 1995; Watkins et al., 2002; MacDermot et al., 2005; Shriberg et al., 2006). However, oromotor and broad cognitive deficits have been claimed to exist as well in people bearing pathogenic mutations of *FOXP2* (Vargha-Khadem et al., 1995; Watkins et al., 2002). In mice the knockout of *Foxp2* (and the transformation with the human pathogenic variant of the gene) gives rise to structural and functional anomalies in the cerebellum (Shu et al., 2005; Groszer et al., 2008), increases long-term potentiation (LTP) in Purkinje cells, decreases long-term depression (LTD) in the striatum (Groszer et al., 2008), and impairs the ultrasonic vocalizations in the pups (Shu et al., 2005). In turn, the knockdown of *FoxP2* in zebra finch mainly affects the area X of the song circuit (a structure homologous to the striatum), and decreases the accuracy of the imitative processes involved in song learning and shortens the critical period for song learning (Haesler et al., 2004).

In Boeckx and Benítez-Burraco (2014) we speculated about a potential link between *FOXP2* and *RUNX2* via *SIRT1*. We later found out that RUNX2 binds the *FOXP2* promoter in human cells overexpressing *RUNX2* (Kuhlwilm et al., 2013).

#### POU3F2

We begin our discussion of *FOXP2* partners with *POU3F2*, which we briefly mentioned above. The POU3F2 protein binds a specific site within intron 8 of *FOXP2* (Maricic et al., 2013). AMHs bear a derived allele of the binding site which is less efficient in activating transcription than the Neanderthal/Denisovan allele (Maricic et al., 2013). In mice the lack of the homopolymeric amino acid repeats that are characteristic of mammalian POU3F2 give rise to a decrease in the rate-limiting enzymes of dopamine and serotonin synthesis in various brain areas, and ultimately, to an impairment of pup retrieval behavior (Nasu et al., 2014). The POU3F2 factor also regulates the upper-layer neuronal migration and identity during the development of the neocortex (McEvilly et al., 2002; Sugitani et al., 2002). A region surrounding *POU3F2* is a risk locus for bipolar disorder (Mühleisen et al., 2014). Moreover, sequence and copy number variations affecting *POU3F2* have been found in patients with developmental and language delays, intellectual disability, schizophrenia, or autism spectrum disorders (Huang et al., 2005; Potkin et al., 2009; Lin et al., 2011). *POU3F2* shows a frontal to temporal gradient patterning in the developing human neocortex and is associated with human accelerated conserved non-coding sequences (Miller et al., 2014). Additionally, POU3F2 interacts with ASCL1, a protein that regulates the development of GABAergic neurons (more on this gene below) and PQBP1 (Waragai et al., 1999), a protein involved in neurite growth and neuron projection (Wang et al., 2013a). *PQBP1* has been linked to developmental delay and microcephaly (Li et al., 2013) and to intellectual disability (Wang et al., 2013a).

#### CNTNAP2

*CNTNAP2* is a candidate for SLI and a FOXP2 target (Vernes et al., 2008). The gene has been also related to different forms of language delay and language impairment (Petrin et al., 2010; Sehested et al., 2010), to autism (Alarcón et al., 2008; Bakkaloglu et al., 2008), and to intellectual disability (Gregor et al., 2011). *CNTNAP2* encodes a neurexin involved in synaptogenesis (Dean et al., 2003), which contributes to the establishment of interconnection patterns within the frontal lobe (Scott-Van Zeeland et al., 2010). In *Drosophila* Cntnap2 (known as NrxIV) functions in midline repulsive axon guidance in conjunction with Robo and Slit (NrxIV physically associates with Robo and Slit, and interactions between NrxIV and Slit are affected in *NrxIV* mutants; Banerjee et al., 2010). According to Banerjee et al. (2010), NrxIV is essential for proper Robo localization and identify Nrx IV as a novel interacting partner of the Slit/Robo signaling pathway. Interestingly, the human CNTNAP2 shows a fixed change (Ile345Val) compared to the Denisovan protein.

CNTNAP2 and a protein called NFASC are components of the nodal region of myelinated fibers (Brown et al., 2001). NFASC is a cell adhesion protein involved in mechanisms of neural plasticity such like neurite outgrowth or the formation of postsynaptic components and the organization of the axon initial segment and nodes of Ranvier during early development (Kriebel et al., 2012). Specifically, loss of the Nfasc factor in adult Purkinje neurons provokes loss of neuron activity and neuron disorganization, and eventually, ataxia (Buttermore et al., 2012). Interestingly, NFASC shows as well a fixed change (T987A) in AMHs compared to Neanderthals/Denisovans (Pääbo, 2014; Table S1).

The expression of *CNTNAP2* in frontostriatal systems is interesting too in light of the pathology of *SRPX2* mutations in the perisylvian cortex. *SRPX2* is another of FOXP2 targets (Roll et al., 2010). Mutations in *SRPX2* give rise to developmental verbal dyspraxia or to congenital bilateral perisylvian polymicrogyria (Roll et al., 2006).

#### FOXP1

The interaction between FOXP2 and FOXP1 is also worth considering in the present context. FOXP1 is a partner of FOXP2 (both proteins physically interact to form heterodimers; Li et al., 2004). Mutations affecting *FOXP1* gives rise to intellectual disability, autism, and language impairment (Hamdan et al., 2010; Horn et al., 2010). In mice both *Foxp1* and *Foxp2* are highly expressed in the developing and mature basal ganglia. *Foxp1* is also expressed in layers III-vof the cortex, the hippocampus, and thalamus (Ferland et al., 2003). Both *Foxp1 and Foxp2* are expressed following neuronal migration, suggesting a role in postmigratory neuronal differentiation (Ferland et al., 2003).

Importantly for our hypothesis, *FOXP1* is expressed along with *RUNX1* and *RUNX3* (two other members of the RUNX family, which are involved in the evolution of sophisticated sensory systems in higher vertebrates) in structures relevant to cortico-laryngeal connections (Inoue et al., 2008). As Inoue et al. (2008) review, *RUNX3* has been shown to be upregulated in autism. The gene is also essential for the target-specific axon path finding of some dorsal root ganglion neurons, and it contributes to specify the termination pattern of sensory axons in the developing spinal cord (Inoue et al., 2008). It is thought to be essential not only for the functional glossopharyngeal system (swallowing), but to play a role in the forebrain as well, including language and social regions of brain (Inoue et al., 2008). *Runx3* knockout mice show severe motor dis-coordination (Inoue et al., 2008).

As for RUNX1, it is synthesized in specific populations of somatic motor neurons in the spinal cord and in cholinergic branchial and visceral motoneurons in the hindbrain. Disruption of *Runx1 i*n mice results in massive neuronal apoptosis (Inoue et al., 2008). Both Runx1 and Runx3 are acetylated by p300 (another gene of interest for us: see below), and these modifications are important for the control of transcriptional activity and protein stability (Jin et al., 2004; Yamaguchi et al., 2004).

#### NKX2-1

*NKX2-1* is another gene potentially linking our initial gene set and the *FOXP2* network. *NKX2-1* plays a key role in the development of the basal ganglia. Hence, one of the two major subpopulations of GABAergic projection neurons in the basal ganglia (with descending projections to the subthalamus and substantia nigra) originates from progenitors expressing *Nkx2-1* (Medina et al., 2014). Medina et al. (2014) also hypothesize that the novel expression of *Nkx2-1* in the subpallium constitutes a major event in telencephalic evolution, and relate it to *Shh* expression and changes in the regulatory region of *Nkx2-1* (*SHH* is a member of our initial gene set of interest). Moreover, the mutation of *NKX2-1* gives rise to benign hereditary chorea, a clinical condition that not only involves hypotonia and chorea, but also learning difficulties in the adulthood (Gras et al., 2012). Interestingly, Foxp2 modulates Nkx2-1 DNA-binding and transcriptional activity, at least in the lung (Zhou et al., 2008; Yang et al., 2010a).

#### CITED2

In our previous work (Boeckx and Benítez-Burraco (2014)) we also highlighted that according to Prüfer et al. (2014) a highly disruptive intergenic change near *CITED2* is 99% derived in AMHs and ancestral in both Altai Neanderthals and Denisovans. We also pointed out that this gene is highly expressed in the mediodorsal nucleus of the thalamus and that it is a regulatory target of FOXP2 (Vernes et al., 2011; Nelson et al., 2013). As we pointed out above, CITED2 is a functional partner of LHX2. Both *Cited2* and *Runx2* are regulated by Tgf (Luo et al., 2005). Moreover, CITED2 is also involved in the establishment of left-right axis (Preis et al., 2006). In mice Cited2 has proved to impact left-right patterning through interactions with the Bmp signaling and Nodal (Lopes Floro et al., 2011). *BMP*s are among the genes encompassing our core set of genes and *NODAL* is a robust candidate for the establishment of bilateral symmetry in the embryo (Zhou et al., 1993; Lowe et al., 1996; Gebbia et al., 1997). Interestingly, CITED2 may play some important role in craniofacial development too (Bhattacherjee et al., 2009). Additionally, Cited2 is a co-activator of Crebbp and Ep300, which play important roles in the initial development of the dorsal neural folds (Bhattacherjee et al., 2009).

#### CREBBP and EP300

Both *CREBBP* and *EP300* are linked to our initial gene set of interest. In osteoblasts the CREB/CBP complex controls the RUNX2-mediated activation and expression of *BMP2* (Shim et al., 2012). Interestingly, both *EP300* and *CREBBP* are candidates for different subtypes of Rubinstein–Taybi syndrome, a complex condition characterized by mental and growth retardation and skeletal abnormalities (Viosca et al., 2010). Individuals with *EP300* mutations have less severe mental impairment, but exhibit more severe microcephaly, and a greater degree of changes in facial bone structure (Hennekam, 2006). In this syndrome main abnormalities at the brain level occur in the rolandic area (Sener, 1995). In mice the knock-out of *Crebbp* is lethal, but when the gene is specifically deleted in postmitotic neurons of the forebrain, behavioral defects are greatly restricted and emerge mostly in the form of problems in object recognition memory (Valor et al., 2011). In turn, neonatal Crebbp(+/-) mice display perturbed vocalization behavior (Wang et al., 2010b).

**FIGURE 3:**
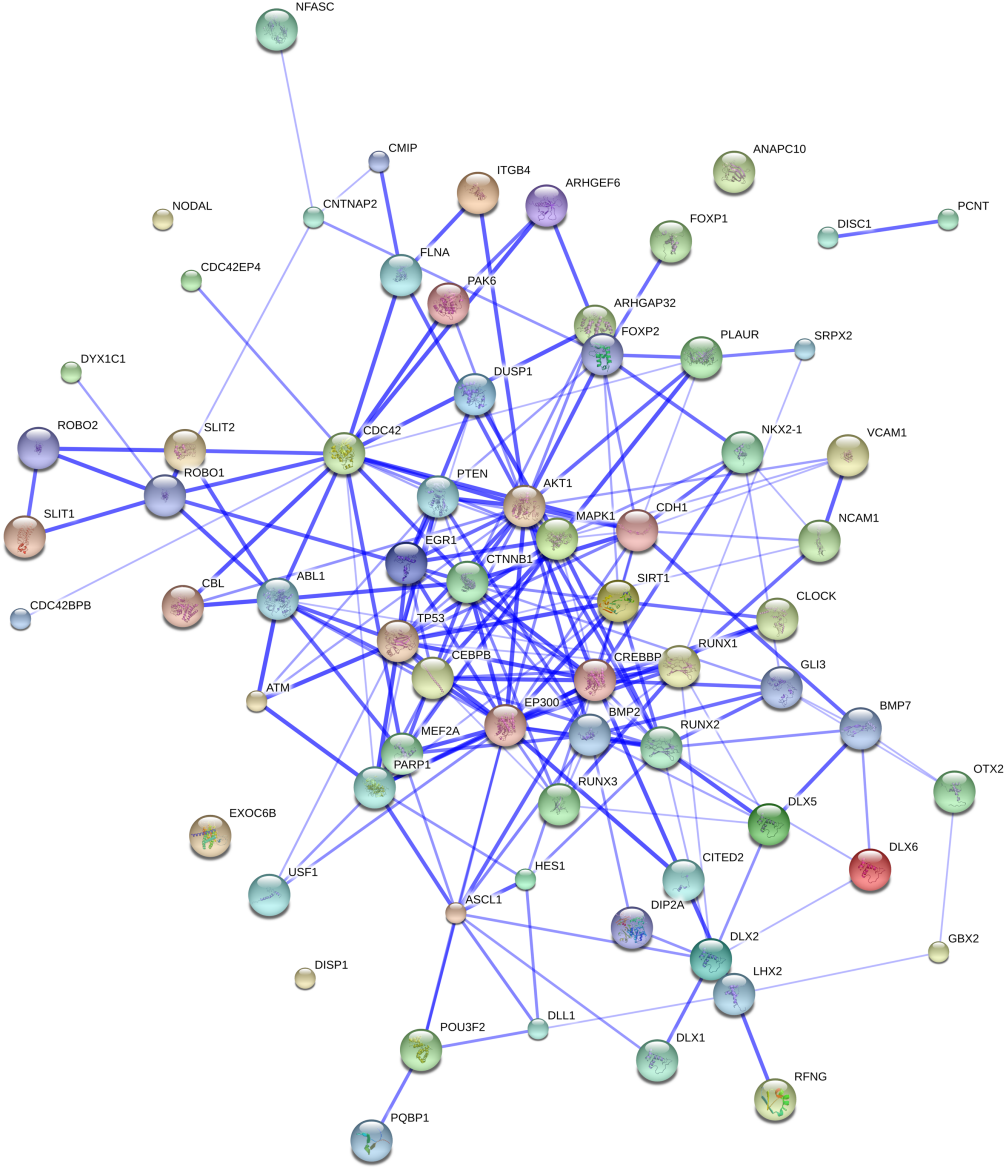
**The whole set of genes discussed in this paper and our initial gene set related to globularity.** The network was generated by String 9.1 with the proteins discussed in Sections “The ROBO/SLIT Suite,” “*FOXP2* and Partners,” and “Other Genes of Interest” plus the gene set related to globularity as advanced in Boeckx and Benítez-Burraco (2014). The medium confidence value was 0.0400. The caveats noted for **Figure 1** apply.

Additionally, both *EP300* and *CREBBP* are components linking our initial gene set to the *FOXP2* and *ROBO1* networks (see **Figure 3**). Their involvement in our network led us to search for other genes that are functionally connected to them. Thus, String 9.1 predicts confident links of CREBBP with ASCL1, CDKN1A, NCOA6, SIRT1, YAP1, RUNX1, and CTNNB1. In turn, EP300 is expected to be linked functionally to all those genes, but also to ETV4 and PIN1 (see **Figure 3**). Rather than going through all these genes, we decided to focus on only the most promising ones, for which we found robust links in the literature.

#### ASCL1

To begin with, ASCL1 is one of the proteins that regulate the DLX suite, a core component of our initial gene set in (2014). According to Letinic et al. (2002) 65% of neocortical GABAergic neurons in humans express the transcription factors ASCL1, DLX1, and DLX2 (the latter two are members of our initial gene set), and originate from *ASCL1*-expressing progenitors of the neocortical ventricular and subventricular zone of the dorsal forebrain (there exists a second lineage of neocortical GABAergic neurons, which express *DLX1* and *DLX2*, but not *ASCL1*). Also according to Letinic et al. (2002), modifications in the expression pattern of transcription factors in the forebrain may underlie species-specific programs for the generation of neocortical local circuit neurons, with distinct lineages of cortical interneurons differentially affected in genetic and acquired diseases of the human brain. Moreover, over-expression of *ASCL1* can improve GABAergic differentiation of bone marrow mesenchymal stem cells *in vitro* (Wang et al., 2013b). Together with *Dlx1* and *Dlx2* and the Foxp2 target *Nkx2-1* discussed above, *Ascl1* is involved in the development of the basal ganglia (Anderson et al., 1997; Casarosa et al., 1999). Additionally, *ASCL1* is known to be involved in retinoic acid signaling: interestingly, both FOXP2 and retinoic acid strongly downregulate *ASCL1* (Devanna et al., 2014). Finally, according to Wapinski et al. (2013) ASCL1 recruits POU3F2 during transdifferentiation of fibroblasts to neurons and likely other cell types.

#### DLL1

One partner of *ASCL1* is *DLL1* (see, for example, Nelson and Reh, 2008). *DLL1* is linked to many of the genes involved in vocal learning (Wang, 2011). *DLL1* encodes a member of the NOTCH signaling cascade (actually, DLL1 is a ligand of NOTCH1), which plays a central role in the regulation of neural proliferation and differentiation (Jung et al., 2011). An inverse correlation between oscillations in the Notch effectors Hes1, discussed above, and Dll1 controls neural growth and differentiation (Shimojo et al., 2008). Additionally, *Dll1* may be related to the establishment of asymmetries during development. Hence, mutants for this gene exhibit defects in left-right asymmetry and do not express *Nodal*, the main left-sided determinant (Krebs et al., 2003).

### OTHER GENES OF INTEREST

Some other proteins may help to make more robust the links between our initial gene set and the two gene sets we have reviewed above (RUNX2, ROBO1, and FOXP2 networks, respectively). This is why we discuss them in the remainder of this section.

#### CDC42

Several lines of evidence converge onto *CDC42* to make this gene an appealing one in the context of our hypothesis. According to the Human Brain Transcriptome (HBT) database *CDC42* is highly expressed in the thalamus, with a peak around day 100th and then a slow drop until birth, a significant fact for our (2014) hypothesis, given that globularity is the result of key developmental events in the first year of life. Moreover, Cdc42 is required in pre-migratory progenitors of the medial ganglionic eminence in the ventricular zone for proper cortical interneuron migration (Katayama et al., 2013). Additionally, some partners of *CDC42* are related to cognitive disorders. For example, a type of X-linked intellectual disability is caused by mutations in *ARHGEF6* (and by the concomitant reduction in CDC42 activity), which result in structural anomalies in pyramidal neurons in the hippocampus, a reduction of early phase LTP, and an increase of LTD in some areas of this brain region (Ramakers et al., 2012). Similarly, the disruption of *Pak5* and *Pak6*, which encode two effector proteins of Cdc42 gives rise to learning, memory and locomotion deficits in mice (Nekrasova et al., 2008). Additionally, Cdc42 activity is reduced in living growth cones by the inhibitory axon-guidance cue Slit2 (Myers et al., 2012). Ectopic expression of *Slit2* on glioma cells attenuates cell migration and invasion through inhibition of Cdc42 activity *in vitro*. Moreover, cellular depletion of Robo1 prevents Slit2 inhibition of Cdc42 activity (Yiin et al., 2009).

*CDC42* also allows for further connections between *FOXP2* and *RUNX2* networks. To begin with, FOXP2 regulates the expression of *CDC42BPB*, one effector of CDC42 (Spiteri et al., 2007). Moreover, during osteoclastogenesis CDC42 regulates FLNA function (Leung et al., 2010). *FLNA* encodes an actin-binding protein that regulates reorganization of the actin cytoskeleton and is required for neuronal migration to the cortex (Fox et al., 1998). Mutation in *FLNA* causes periventricular nodular heterotopias (Fox et al., 1998; Sheen et al., 2001). Interestingly, FLNA also binds *CMIP* (Fox et al., 1998), which is one strong candidate for SLI: it may modulate phonological short-term memory, which is a core deficit in SLI, dyslexia, and speech-sound disorder (Newbury et al., 2009). *CMIP* encodes one component anchoring the cellular membrane to the cytoskeleton, and seems to regulate neural migration and/or the assembly of synaptic complexes (Grimbert et al., 2003). FLNA interacts with ITGB4 (Travis et al., 2004), a protein that shows two fixed changes (T1689A and H1748R) in AMHs compared to Neanderthals/Denisovans (Pääbo, 2014; Table S1). Importantly, 1095 bp within the body of the gene *CDC42EP4* (which encodes an effector of CDC42) are hypermethylated in humans compared to Denisovans (consequently, we should expect a lower expression of the gene in AMHs; Gokhman et al., 2014).

Another partner of CDC42, namely, ARHGAP32 bears a fixed change (E1489D) in humans compared to Denisovans (Meyer et al., 2012). *ARHGAP32* encodes a receptor of NMDA which modulates Rho-GTPase activity, thus modulating dendritic spine morphology and strength, and promoting axon growth; moreover, the knockdown of *Arhgap32* in mice results in impaired migration and axonal growth in the developing cerebellar cortex (Kannan et al., 2012). Interestingly, some polymorphisms within the human gene might increase susceptibility for schizophrenia and schizotypal personality traits (Ohi et al., 2012). *ARHGAP32* promotes axon growth downstream of *CDH1* (Kannan et al., 2012). This gene encodes a cadherin involved in the regulation of cell–cell adhesions, mobility and proliferation of epithelial cells. *CDH1* also coordinates cortical neurogenesis and size, to the extent that the mutation of the gene gives rise to microcephaly (Delgado-Esteban et al., 2013). *CDH1* seems to regulate neural connectivity as well, from axon and dendrite morphogenesis and growth to synapse differentiation and remodeling (Konishi et al., 2004; Huynh et al., 2009; Yang et al., 2010b). According to the HBT database the gene is expressed at high levels in the thalamus before birth. One of *CDH1* partners, namely, *ANAPC10* (Nourry et al., 2004) shows signals of a selective sweep in AMHs compared to Altai Neanderthals (Prüfer et al., 2014).

#### CBL

*CBL* encodes a negative regulator of several receptor protein tyrosine kinase signaling pathways (Joazeiro et al., 1999). Mutations in this gene cause Noonan syndrome-like disorder, a condition characterized by facial dysmorphism, a reduced growth, and variable cognitive deficits, among other symptoms (Martinelli et al., 2010). In mice activated Cdc42 prevents Cbl from catalyzing ubiquitination of specific receptors (Wu et al., 2003). Cbl interacts with some other of our genes of interest. Hence, Cbl phosphorylation depends on Abl1 (Miyoshi-Akiyama et al., 2001). Additionally, CBL belongs to the TRAIL pathway, associated with bone metabolism (Zhang et al., 2011). Moreover, Cbl ubiquitinates Notch1, triggering its degradation (Jehn et al., 2002). Finally, *CBL* is located in a region showing signal of a strong selective sweep (20-fold enrichment over random) in AMHs compared to Altai Neanderthals (Prüfer et al., 2014).

#### MEF2A

While discussing the role of *SIRT1* in connection with *FOXP2* and *RUNX2* in our (2014) paper, we were led to consider *MEF2A*. The research we report on here led us back to this gene on numerous occasions. *MEF2A* has been recently implicated in differences between human and chimpanzee prefrontal cortex development (Liu et al., 2012). In addition, as reviewed in Pfenning (2012), *MEF2A* plays a significant role in songbirds. It turns out that *MEF2A* is linked to many of genes we have examined here. To begin with, MEF2A interacts with ASCL1 to modulate the expression of genes that are critical for neuronal differentiation (Mao and Nadal-Ginard, 1996; Gohlke et al., 2008). Additionally, EP300 interacts with MEF2A (He et al., 2011). Moreover, according to the HBT the gene is highly expressed in the thalamus. Finally, String 9.1 returns results where MEF2A is linked to ABL1 (an partner of ROBO1 that we have reviewed above) via ATM (a key controller of cell response to DNA damage and for genome stability that has been associated with ataxia telangiectasia, a condition involving cerebellar degeneration and dysarthria among other many symptoms, although not mental retardation; Ejima and Sasaki, 1998; Laake et al., 2000). According to String 9.1 ATM is also linked to PTEN and (indirectly) to FOXP1.

#### TP53

String 9.1 predicts TP53 to be linked to USF1, which we took to be central in our (2014) paper, but also to SIRT1, CDH1, ASPM (a well-known candidate for microcephaly), and PTEN. *TP53* has been related to schizophrenia (Ni et al., 2005). Moreover, the expression level of the gene in humans is different compared to chimps/rhesus (Konopka et al., 2012). According to the HBT database the gene is expressed in the thalamus (this is the only structure where the gene is upregulated after birth). Additionally, there is a human-specific variant of the protein that bears an Arg in position 72, while Neanderthals/Denisovans exhibit a Pro (this is not a fixed change yet, since Arg72 frequencies range from 20 to 80% in AMHs, the lowest levels being observed among Sub-Saharan peoples; Paskulin et al., 2012).

#### CTNNB1

CTNNB1 is expected to be linked to many of the genes we are interested in, including *RUNX2*, *EP300*, *CREBB4*, *SIRT1*, *BMP2*, *ROBO1,* and *CDC42* (prediction based on String 9.1 data). Specifically, Ctnnb1 binds the *Runx2* promoter and upregulates *Runx2* expression (Han et al., 2014). Additionally, the presence of an active Slit2/Robo1 signal blocks the translocation of Ctnnb1 into the cell nucleus (Chang et al., 2012). As mentioned in Boeckx and Benítez-Burraco (2014), CTNNB1 is a strong candidate for autism. Interestingly, it also interacts with *PCDH11X/Y*, a gene pair that underwent accelerated evolution in our lineage (Williams et al., 2006), and has been linked to cognitive disorders such as schizophrenia (Crow, 2013) and language acquisition delay (Speevak and Farrell, 2011).

#### EGR1

*EGR1* is an immediate early gene that encodes a transcription factor involved in neuronal plasticity needed for consolidation of new memories (Veyrac et al., 2014). In the brain, signaling from synapses to the nucleus of neurons activated during learning tasks induce the expression of this kind of transcription factors, which mediates the gene programs needed for the stable functional and structural remodeling of the activated networks (this allows memory to be later reactivated upon recall). In mice *Egr1* mutants are impaired in long-term (but not short-term) recognition memory (Bozon et al., 2003). Interestingly, in songbirds the expression of *EGR1* is induced by singing in song nuclei, although the gene is also expressed in adjacent brain regions in response to non-vocal motor behaviors, quite contrary to *DUSP1*, which shows a motor-driven expression in the forebrain only in song nuclei and only in vocal learners (*DUSP1* is up-regulated in sensory-input neurons of the thalamus and telencephalon; Horita et al., 2010, 2012). *EGR1* functionally interacts with some of the genes we have examined, including *PTEN* (Kim et al., 2014) and *AKT1* (Hernández et al., 2013). Similarly, EGR1 physically interacts with CBP and EP300 to modulate gene transcription (Silverman et al., 1998). Importantly, *EGR1* is a target of both FOXP2 (Konopka et al., 2009) and RUNX2 (Kuhlwilm et al., 2013). Moreover, EGR1 downregulates *PLAUR* in osteosarcoma cell lines (Matsunoshita et al., 2011). *PLAUR* is a target of FOXP2 (Roll et al., 2010) and encodes an effector of SRPX2, another FOXP2 target (Royer-Zemmour et al., 2008). Finally, EGR1, acting as an effector of SOX9B and being regulated by RUNX3, modulates the BMP signaling needed for cranial cartilage development in zebrafish (Dalcq et al., 2012; *BMP*s are among the genes encompassing our core set of genes). According to the HBT database, *EGR1* is expressed in the thalamus and the cortex: its expression level steadily increases until first year of life.

#### CEBPB

*CEBPB* encodes a transcription factor that binds – the promoter of *EGR1* (Calella et al., 2007). Moreover, the protein CEBPB physically interacts with EGR1 to modulate gene transcription (Zhang et al., 2003). Compound *Cebp* knockout mice (i.e., for genes *Cebpa* and *Cebpb*) show defective differentiation of cortical dendrites (Calella et al., 2007). Transcriptional activation by CEBPB also involves the coactivators CBP and EP300 (Mink et al., 1997; Guo et al., 2001). Interestingly, *CEBPB* also plays a role in osteogenesis. Hence, Cebpb is a key modulator of *Runx2* expression in bone formation, specifically during chondrocyte (Hirata et al., 2012) and osteoblast (Gutiérrez et al., 2002) formation. Deletion of *Cebpb* gives rise to suppressed differentiation of osteoblasts and delayed chondrocyte hypertrophy, thus postponing bone formation (Tominaga et al., 2008) Ultimately, *CEBPB* is claimed to be a candidate for cleidocranial dysplasia (Huang et al., 2014). *CEBPB* is also a target of FOXP2 (Konopka et al., 2009).

#### NCAM1

*NCAM1* encodes a protein involved in cell-to-cell interactions that plays a key role in the development and differentiation of the brain (Fujita et al., 2000; Prag et al., 2002) The primary transcript of *NCAM1* is modified both posttranscriptionally (alternative splicing of the mRNA generates three main protein isoforms and the ectodomain shedding of NCAM1 isoforms can produce an extracellular soluble neural cell adhesion molecule fragment) and posttranslationally (a residue of polysialic acid is added to the molecule) following developmental cues (Rutishauser and Goridis, 1986; Wang et al., 1998; Cox et al., 2009). Moreover, the gene is subject to epigenetic modifications that affect its splicing pattern (Schor et al., 2013). At the brain level *NCAM1* plays a pivotal role in axonal and dendritic growth and synaptic plasticity, and ultimately, in cognition (Rønn et al., 2000; Hansen et al., 2008). Hence, *Ncam1*-deficient mice are impaired in working/episodic-like memory performance (Bisaz et al., 2013). Alterations in *NCAM1* expression and/or proteolytic cleavage of the protein have been related to different neuropsychiatric conditions, including schizophrenia, bipolar disorder and Alzheimer’s disease (Atz et al., 2007) and may contribute to the cognitive dysfunctions observed in these diseases. Specifically, the amount of the NCAM1 extracellular proteolytic cleavage fragment has been reported to be increased in schizophrenics (Vawter et al., 2001). In mice when this fragment is overexpressed GABAergic innervation is impaired and the number of dendritic spines on pyramidal neurons in the prefrontal cortex becomes reduced. In turn this results in the impairment of long- and short-term potentiation in the prefrontal cortex, although synaptic plasticity is normal in the hippocampus (Brennaman et al., 2011). Interestingly, the absence of polysialic acid in the protein gives rise to misguidance of thalamocortical fibers and deficiencies of corticothalamic connections (Schiff et al., 2011). *NCAM1* is functionally linked to some of the genes we considered. Hence, RUNX1 controls the expression of *NCAM1* (Gattenloehner et al., 2007). Moreover, *NCAM1* is a putative target of both RUNX2 (Kuhlwilm et al., 2013) and FOXP2 (Konopka et al., 2009).

#### VCAM1

NCAM1 interacts to VCAM1, one of the proteins showing a fixed change (D414G) in AMHs compared to Neanderthals/Denisovans (Pääbo, 2014; Table S1). VCAM1 is a cell surface glycoprotein involved in cell adhesion. In the adult forebrain, subventricular zone neurons arise from type B neural stem cells, which are anchored by specialized cells expressing high levels of VCAM1. Disruption of *VCAM1* disturbs the architecture of the subventricular zone and increases neurogenesis in some areas (specifically, in the olfactory bulb; Kokovay et al., 2012). Interestingly, *VCAM1* is upregulated by CLOCK (Gao et al., 2014), which interacts with RUNX2 (Reale et al., 2013), a link we reviewed in our paper (2014). According to Shimomura et al. (2013), Usf1, a member of our initial gene set, is able to compensate *Clock* mutations in mice, this ultimately suggesting that Usf1 is an important modulator of molecular and behavioral circadian rhythms in mammals. Also *DUSP1* is upregulated by CLOCK (Doi et al., 2007).

#### MAPK1

MAPK1 is a potential hub linking many of our genes of interest. MAPK1 regulates the transcription of FOXP2 target *PLAUR* (Lengyel et al., 1996). Moreover, MAPK1 and DUSP1 physically interact at the brain level, since DUSP1 dephosphorylates MAPK1 (among other MAPK proteins; Choi et al., 2006; Lomonaco et al., 2008). Additionally, MAPK1 is a positive regulator of *RUNX2* (Lee et al., 2011). Overall, MAPK1 seems to play an important role in osteogenesis. Specifically, inhibition of MAPK1 activity leads to significant decrease in BMP9-induced osteogenic differentiation and bone formation (Zhao et al., 2012). Moreover, BMP2 [also a member of our (2014) set] induces osteoblastic differentiation by a DUSP1–MAPK1 dependent mechanism (Ghayor et al., 2009). MAPK1 plays a key role in cognition and brain function too. The gene is required for neuronal cell fate determination. In mice deletion of *Mapk1* results in a reduction in cortical thickness and mutant mice for *Mapk1* exhibit important deficits in associative learning (Samuels et al., 2008). Additionally, a stimulus-dependent increase of Mapk1 signaling resulting from the ablation of *Erk1* gives rise to a strong enhancement of striatum-dependent long-term memory, and ultimately, to a modification of the long-term adaptive changes underlying striatum-dependent behavioral plasticity (Mazzucchelli et al., 2002). In humans microdeletions on chromosome 22q11.2 encompassing *MAPK1* give rise to microcephaly, impaired cognition, and developmental delay (Samuels et al., 2008). Actually, there exists a group of genetic disorders (including Costello syndrome, Leopard syndrome, and Noonan syndrome) that are caused by mutations in upstream elements of the MAPK signaling cascade. Among the distinctive symptoms, one finds craniofacial defects, developmental delay, and mental retardation (see Bentires-Alj et al., 2006, for a review). Similarly, the mutation of downstream elements in the MAPK cascade has been associated with mental retardation (Weeber and Sweatt, 2002).

#### ‘Dyslexia’-related genes (beyond ROBO1)

In addition to *ROBO1*, other potential candidates for dyslexia (according to Poelmans et al., 2011) are linked to some of the genes we considered in the context of globularity in our (2014) article. Thus, DIP2A functions as a transcriptional co-activator of *DLX2* and *DLX5*, and plays an important role in the development of the basal ganglia (Yu et al., 2001). Moreover, BMP2 blocks the binding of DIP2A to a protein called FRP (Tanaka et al., 2010), a member of the Wnt signaling pathway and a target of PAX6 during the regulation of axonal connectivity; in turn, Pax6 mediates the response of growing axons to Sfrp1 (Sebastián-Serrano et al., 2012), which functions as an enhancer of the Wnt/PCP signaling in dopamine cells and a regulator of Wnt/PCP-dependent functions in midbrain development (Kele et al., 2012).

Another potential candidate for dyslexia, *PCNT*, encodes pericentrin, a protein of the centrosome, which interacts with DISC1, the product of one robust candidate for schizophrenia (Miyoshi et al., 2004). *DISC1* is a target of FOXP2 too (Walker et al., 2012). *PCNT* is cited by Green et al. (2010) among the 11 genes that show non-synonymous and non-fixed substitution changes in their coding sequences compared to Neanderthals. Moreover, the mutation of the gene gives rise to a condition called microcephalic osteodysplastic primordial dwarfism type II, characterized by different bone abnormalities and by a reduced brain size but usually with near-normal intelligence (Rauch et al., 2008).

Finally, among the genes that are differentially expressed among (mammalian) vocal learners (according to Wang, 2011), one finds *PARP1* that regulates the dyslexia-susceptibility gene *DYX1C1*, important for neuronal migration in the developing cortex (Tapia-Páez et al., 2008).

## CONCLUSION AND PROSPECTS

Boeckx and Benítez-Burraco (2014) put forth a hypothesis concerning the emergence of the language-ready brain that highlighted the potential role of a small set of genes such as *RUNX2*. Our original focus was on what one may want to call the syntax-semantics branch of our language faculty. The other branch, ‘the externalization component,’ crucial to convey the syntactically coded meanings of sentences, was left for future research. Our goal in this paper has been to attend to that aspect of language, and refine the gene set we originally put forward by taking into account what we know about vocal learning. In the vocal learning literature two neural components are often presented as critical: the direct cortico-laryngeal connection and the cortico-thalamo-basal ganglia pathway, and genes like *ROBO1* and *FOXP2* have been associated with these networks. The purpose of this paper has been to see if points of contact and potential functional links could be hypothesized between *ROBO1*, *FOXP2*, and their partners, and our initial gene set. Identifying such potential links would offer a much more comprehensive picture of our hypothesis liking globularity to language-readiness, opening up new ways to falsify it. Eventually, the genes we have advanced in this paper may be regarded as potential objects of inquiry for future research on the genetic underpinnings of language and of language disorders.

It stands to reason that as we learn more about each gene discussed here, the potential links between the various networks are likely to grow, so we are aware that we are just beginning to scratch the surface of a big puzzle. We are also aware of the limitations of attempts like ours to perform literature-based assembly of protein–protein and gene-regulatory networks. For this reason we think it fit to conclude this paper by stressing the need to test the robustness of each of the connections hypothesized here. Finding a transcription factor ChIP peak in the vicinity of a gene is not enough to conclude that the gene is regulated by the transcription factor. This interaction has to be demonstrated *in vitro* and/or *in vivo*. Likewise, finding that some protein has acquired a fixed coding change in AMHs compared to Neanderthals/Denisovans does not always entail that the change has impacted on protein function. This impact has to be demonstrated empirically. Similarly, although we have focused on the strongest links found in the literature, eventually these links (often binary) have to be properly evaluated in order to know if they are actually biologically significant and meaningful with respect to the phenotype of interest. We also acknowledge that the literature and the datasets we have relied on may be incomplete or biased because of the unavoidable focus on some genes as opposed to others. One should not forget that absence of evidence is not evidence of absence regarding protein–protein or DNA-protein interactions. It is also clear that the attested links for intensely studied proteins are always be more salient and more numerous than for less studied proteins. The same holds for disorders: some have been intensively examined, while others remain poorly described. So, we don’t want to exaggerate our results. Still, we hope that the current study can serve as a useful starting point for future investigations that regard our initial hypothesis as promising.

We would be delighted if the information we provided here help constrain the search space of future work.

To this end, we close with a few suggestions concerning how the expanded hypothesis presented here could be used and tested experimentally.

The most urgent task consists in showing that all the genes examined really interact in the human brain. In many cases evidence for this interaction comes from knockout experiments involving mice or from expression assays in cell lines. ChiP experiments are a good first approximation, but the results of these experiments need to be further refined. Additionally, we need to determine the exact anatomical and functional consequences of the mutation of all these genes in humans and/or of changes in their expression levels, with a special focus on the brain areas involved in language processing. We think that it would be extremely valuable to examine these consequences in non-human vocal learners, for which we can already rely on a fair amount of knowledge. Knockdown experiments could be conducted in vocal learners in order to test whether these enhanced networks centered on FOXP2 and ROBO1 and connected to RUNX2 really account for key aspects of externalization circuits. Finally, we need to check whether the fixed changes (or even the changes under selection) in the human proteins (compared to other hominins) had structural and functional consequences. This should also be complemented with additional examination of archaic genomes, with a special focus on changes in the regulatory regions of genes (promoters, enhancers, etc.) and in genes that do not encode proteins (e.g., ncRNAs). All of this will help us provide fuller accounts of how our species came to be language-ready.

## Conflict of Interest Statement

The authors declare that the research was conducted in the absence of any commercial or financial relationships that could be construed as a potential conflict of interest.

## Footnotes

1 http://www.ncbi.nlm.nih.gov/pubmed

2 http://human.brain-map.org/microarray/search

3 http://www.brainspan.org/lcm/search/index.html

4 http://www.brainspan.org/rnaseq/search/index.html

5 http://www.ncbi.nlm.nih.gov/omim/

6 http://string-db.org/
